# Stability and Reusability of Tungsten Catalyst on Structured Support in Catalytic Ozonation of Textile Wastewater

**DOI:** 10.3390/molecules30040969

**Published:** 2025-02-19

**Authors:** Aleksandra Kędzierska-Sar, Maciej Fronczak, Marta Gmurek, Lucyna Bilińska

**Affiliations:** 1 Department of Molecular Engineering, Faculty of Process & Environmental Engineering, Lodz University of Technology, Wolczanska 213, 90-924 Lodz, Poland; maciej.fronczak@p.lodz.pl (M.F.); marta.gmurek@p.lodz.pl (M.G.); lucyna.bilinska@p.lodz.pl (L.B.); 2 Biliński Factory of Colour, Mickiewicza 29, 95-050 Konstantynow Lodzki, Poland

**Keywords:** tungsten catalyst, catalytic ozonation, thin-layer plasma catalyst, textile wastewater, reusability

## Abstract

Since heterogeneous catalytic ozonation (HCO) has become a leading trend in advanced oxidation processes, finding new prospective catalysts has become crucial. Plasma-enhanced chemical vapor deposition (PECVD) is a method of thin-layer deposition that is useful in catalyst production on structured supports. This study presents a novel tungsten (W)-based catalyst used in HCO for textile wastewater discoloration. By changing PECVD parameters, we were able to design and prepare several types of diverse catalysts in terms of morphology and composition. Energy-dispersive X-ray spectroscopy was used for catalyst characterization and revealed a nano-sized granular morphology. The catalyst thickness was below 500 nm, preserving the geometry of the support. The satisfactory high W catalyst activity in dye removal was investigated through a catalytic test. The increased speed in color removal, represented by the enhancement factor, was equal to 1.47 when comparing single and catalytic ozonation. A high and almost unchanged color removal efficiency was maintained over seven cycles of HCO, allowing for more than 5 h of successful use.

## 1. Introduction

The textile industry is one of the most severe polluters, with thousands of cubic meters of highly polluted wastewater being generated annually, posing a significant environmental threat. Textile finishing operations such as washing, dyeing, and printing introduce tons of chemicals into the water environment. Consequently, substances such as waxes, softeners, surfactants, and dyes that are difficult to degrade are released into water bodies through textile wastewater drains [1].

Most textile auxiliary agents exhibit an unfavorable biodegradability index (BI = Biochemical Oxygen Demand (BOD) /Chemical Oxygen Demand (COD)), which is far lower than the 0.4 threshold, the limiting value. A low BI makes textile wastewater difficult to treat in biological wastewater plants [1,2]. Therefore, advanced oxidation processes (AOPs) are considered promising alternatives for textile wastewater treatment [2]. AOPs use hydroxyl radicals (^•^OH) as powerful oxidants to degrade problematic pollutants, such as dyes, making them more amenable to biotreatment and less environmentally harmful [3]. In particular, heterogeneous catalytic ozonation (HCO) has shown to be an attractive method for textile wastewater treatment, making it a leading trend in AOPs. Therefore, finding new catalytic materials that are both active and stable for HCO has become a new focus within AOP research.

A significant trend is to find less expensive alternatives to noble metals. One prospective catalytic material is tungsten (W), a d-block metallic element. The name originates from Swedish, meaning “heavy stone” (tung—heavy; sten—stone). W is characterized by a high density of 19.3 g/cm³, which is close to that of gold (19.32 g/cm³) [4,5]. W-based catalysts can be found in various forms, such as W oxides, sulfides, carbides, and heteropolytungstates [4]. However, W oxides are the most commonly encountered. A structural analysis (e.g., X-ray photoelectron spectroscopy, XPS) reveals multiple oxidation states (2+, 3+, 4+, 5+, 6+) in oxidized W-based materials, but W typically forms WO_3_. W trioxide exists in several allotropic (crystalline) forms, including two monoclinic structures: α-WO_3_ (yellow oxide) and γ-WO_3_ (violet oxide); a triclinic structure β-WO_3_ (blue oxide); and a hexagonal form (h-WO_3_) [6,7]. W dioxide is another example, with a rutile-like oxide structure. W oxides can form structures with other support materials such as TiO_2_, Al_2_O_3_, SiO_2_, etc. [4,8]. All of these structures play significant roles in catalysis.

W has been investigated in numerous catalytic processes. WO_3_ is useful in dehydration, dehydrogenation, and condensation, making it a valuable catalyst in chemical processing [9]. One example of W oxide’s catalytic activity is its use in the catalytic dehydration of methanol to dimethyl ether [10]. The role of W materials in environmental applications has been rising in recent years. Currently, W-based catalysts, including WO₃, are widely used in applications such as DeNOx (the reduction of nitrogen oxides (NOₓ) emissions), CO₂ capture, pollutant oxidation in gas and liquid phases, and gas sensors [4,11,12]. However, the wide bandgap (2.6–3.0 eV) of WO_3_ makes it a semiconductor, which can be light-excited with radiation corresponding to visible light, enabling its use as a photocatalyst in environmental applications [4].

In one of the first works on W-based catalytic ozonation, Nishimoto and co-workers used WO_3_ as a visible-light-responsive catalyst in phenol mineralization [13]. They showed that WO_3_ produces photoexcited electrons that mediate ozonide radical (O_3_^•−^) activation. Since then, WO_3_ has been widely used in photocatalytic ozonation. Later, Rey and co-workers enhanced the photocatalytic activity of WO_3_-based materials by decomposing ibuprofen, promoting electron transport on the catalyst surface of the monoclinic structure where oxygen vacancies were present [14].

Photocatalytic ozonation in the presence of WO_3_ for dye removal remains a relatively under-researched topic. Although studies on dye photocatalysis mediated by WO_3_ have been reported [4,11], the catalytic efficiency of WO_3_-based materials in dye ozonation has only recently received attention in some studies [15,16,17].

In contrast to WO_3_, tungsten carbide (WC) has been less investigated, but it is a promising material in catalysis. WC is a transition metal carbide (TMC) material, similar to other metal carbides such as molybdenum carbides, and is characterized by specific steric and electronic properties that arise from the incorporation of carbon atoms into the metal lattice [18,19]. WC has proven to be an excellent catalyst in hydrogenolysis [18,20], hydrogenation [21,22,23], isomerization [24], deoxygenation [25,26], reforming [27,28], and H_2_ evolution reactions [29,30,31].

Carbon materials, including carbides, play a significant role as catalysts in catalytic ozonation [32,33]. However, to the authors’ knowledge, there is a gap in research on the use of metal carbides, and WC has not been applied in HCO to date.

This work investigates the use of W-based materials, including W oxides and WCs, as potential catalysts in the catalytic ozonation of textile dyes. Plasma-enhanced chemical vapor deposition (PECVD), a specialized technique for creating thin-film materials, was used to produce W-based catalysts. The material characteristics were analyzed using scanning electron microscopy (SEM) and XPS. The catalytic activity and stability in the ozone treatment were evaluated through the removal of the dye Reactive Black 5 (RB5), using both serial and cyclic tests.

## 2. Results and Discussion

The study proceeded in four main stages: catalyst preparation, catalytic testing, characterization of the catalytic material after use, and then the application of the most promising catalyst in real wastewater treatment. The first stage focused on how PECVD conditions, followed by post-treatment, influenced the catalyst’s characteristics. The second stage involved testing the catalyst’s activity test and assessing its reusability in cyclic dye removal from textile wastewater. In the third stage, material analysis of the catalyst after use provided insights into its stability. The fourth stage evaluated dye removal in real wastewater, examining kinetics, COD, and toxicity.

### 2.1. Catalyst Characterization

**Figure 1:**
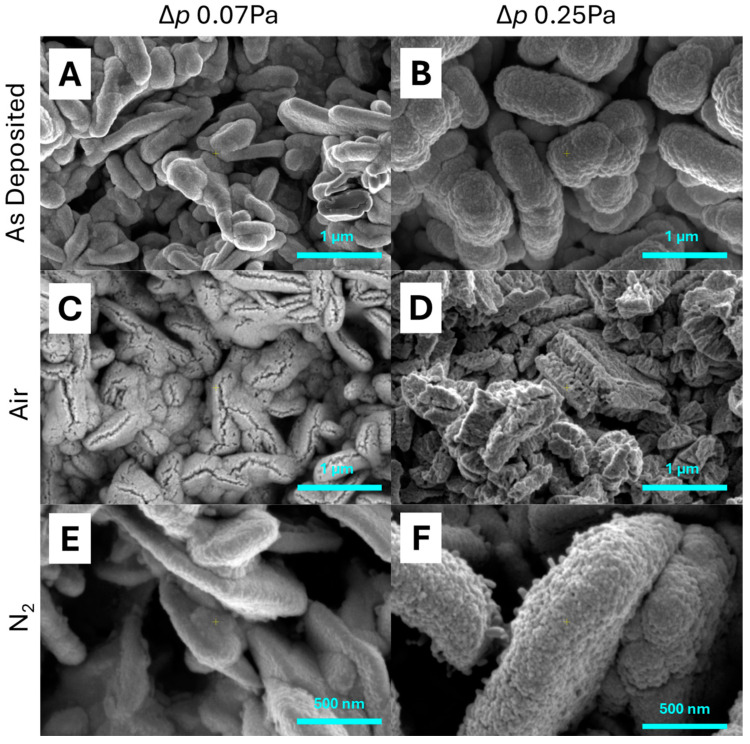
SEM images of microstructure the W-based catalysts deposited through PECVD with 80 W power and two differential pressures of the precursor gas, and different post-treatment (AsD, Air, N_2_): Δ*p* = 0.07 Pa (**A**,**C**,**E**) and Δ*p* = 0.25 Pa (**B**,**D**,**F**).

All catalyst types were prepared by PECVD. Figure 1 shows the SEM micrographs of the catalyst surface types depending on deposition parameters (80 W of glow discharge power and two differential pressures of the precursor gas: Δ*p* = 0.07 Pa and Δ*p* = 0.25 Pa) and post-treatment (AsD—as-deposited samples (without post-treatment), Air—oxidized through calcination in air atmosphere, N_2_—pyrolyzed in nitrogen atmosphere).

Samples presented in Figure 1 are characterized by visibly varied morphology depending on deposition parameters. When the differential pressure of the precursor gas was lower and equal to Δ*p* = 0.07 Pa, the structure was of a more lamellar type built vertically relative to the surface of the support. At the same time, the small amount of the deposited film did not fully change the needle-shaped structure of the prepared Kanthal support through calcination (the support characterized is elsewhere [34]). When the differential pressure of the precursor gas was higher and equal to Δ*p* = 0.25 Pa, the morphology was of granular type with a visibly thicker film. Moreover, the change in deposition parameters increased the thickness. The thickness of the film deposited with a lower precursor stream (Δ*p* = 0.07 Pa) was below 100 nm, while a higher precursor stream (Δ*p* = 0.25 Pa) resulted in a thickness of up to 350 nm. Therefore, the thickness increases as the precursor stream increases, while the discharge glow power remains constant. However, all deposited films remained far below 1 µm and are considered as “thin films.”

After calcination in an air atmosphere, the sample morphology changed to a more crystalline form. Cracks or voids within the deposited film arose—mostly along the thinner side of the alumina support. It can be assumed that the growth of the crystalline structure of WO_3_ (the calcination temperature of 600 °C is appropriate for WO_3_ formation in the air atmosphere [35]) is the cause of such changes. The changes were more easily observed in the thicker film, because the thin film changed its morphology from oval shapes to more angular ones.

Pyrolysis in nitrogen did not drastically change the morphology of samples deposited at 0.07 Pa and 0.25 Pa; however, there were some minor differences. The main presence of needles, which grew from the original layer, is important. They could be observed more easily for the 0.25 Pa deposition pressure, but they were also present for the lower-pressure sample (0.07 Pa). This kind of structure could be recognized as lower W oxides WO_2.9_ or, more probably, WO_2.7_ due to their monoclinic structure [6].

According to XPS, the atomic composition also depended on the samples’ deposition parameters just after PECVD. Table 1 shows the W, C, and O content in AsD samples and after Air and N_2_ treatment. Low W content, below 20%, was observed for 0.07 Pa and 0.25 Pa precursor differential pressure, except for the AsD sample at 0.25 Pa precursor pressure, where its amount reached a level of 23 at.%. Consequently, oxygen and carbon were higher and equal to 30% and 56%, respectively, for the lower precursor stream. Their atomic content decreased with the precursor stream growth.

The calcination in an air atmosphere resulted in a higher oxygen amount; surprisingly, a relatively high amount of carbon was still present in the sample (56 at.% vs. 41 at.% in AsD 0.07 Pa). The W to C ratio was equal to 0.31, and W to O was 0.26. For the higher differential pressure—0.25 Pa—and further calcination in air, the sample contained more oxygen, up to 61 at.%, and the carbon was much less.

After pyrolysis in a nitrogen atmosphere, the difference between 0.07 Pa and 0.25 Pa differential pressures on atomic composition was easy to notice. For lower differential pressure, after calcination, only a small amount of carbon was removed, and there was still oxygen in the sample. The W/C ratio increased from 0.24 to 0.32, and W/O remained almost unchanged from 0.45 to 0.43. In the case of differential pressure 0.25 Pa, the tendency was the opposite. The amount of carbon decreased—W/C ratio increased from 0.55 to 0.63. Contrary, the W/O ratio decreased by half—from 0.7 to 0.32.

**Figure 2:**
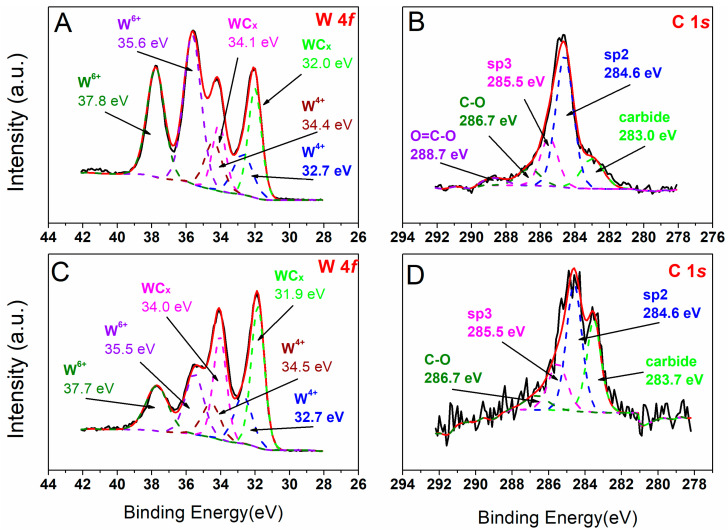
XPS deconvolution for W4f (**A**,**C**) and C1s (**B**,**D**) positions for the AsD samples at Δ*p* = 0.07 Pa (**A**,**B**) and 0.25 Pa (**C**,**D**).

Surface composition, chemical states, and the relative contributions of each component were evaluated based on the deconvoluted high-resolution spectra. The spectra of C1s and W4f for AsD samples at both 0.07 Pa and 0.25 Pa differential pressure are shown in Figure 2. It can be seen that the W atoms were in oxidized form—WO_3_ (35.5 ± 0.1 eV) and WO_2_ (32.7 ± 0.1 eV), but also as a carbide form (31.9 ± 0.1 eV).

The carbide content increased with the differential pressure during deposition. At 0.07 Pa, 27.4% of W was in carbide form, whereas at 0.25 Pa, this value nearly doubled to 47.3% (Table 2). Calcination in air at 600 °C resulted in pure WO_3_ for both pressures. Pyrolysis in a nitrogen atmosphere at 850 °C caused only a minor reduction of WO_3_ to WO_2_, with no change in carbide content for the 0.07 Pa sample. In contrast, for the 0.25 Pa sample, nitrogen treatment resulted in the formation of metallic W, a substantial increase in WO_3_, and a reduction of carbides to 11%. The observed behavior differences may be related to the thickness of the deposited layer, which could limit the gas availability on the surface. Another possible reason could be the reduced carbon content compared to the as-deposited sample, indicating the removal of carbon as CO or CO_2_, which could act as reducing/oxidizing agents [36].

The C1s spectrum revealed peaks corresponding to carbides (283 ± 0.7 eV), adventitious carbon (sp^2^, 284.6 ± 0.1 eV), C–C/C–H (sp^3^, 285.5 ± 0.1 eV), C–O (286.7 ± 0.1 eV), and C=O (288.7 ± 0.1 eV), with varying proportions. In the AsD samples, most carbon was in the form of sp^2^ carbon, indicative of adventitious carbon. The carbide carbon accounted for 15% and 30% for 0.07 and 0.25 Pa, respectively. A stable amount of C–C/C–H (19%) was present in both samples. Calcination in air resulted in a significant increase in adventitious carbon, up to around 80%, with sp^3^ carbon and a small amount of oxidized C–O observed for 0.07 Pa. Nitrogen treatment for 0.07 Pa reduced the amount of carbon bonded with W, favoring the oxidized form of carbon. In contrast, the carbon bonded with W more than doubled in the 0.25 Pa sample after nitrogen treatment, and the sp^2^ carbon content decreased.

### 2.2. Catalytic Test Results

**Figure 3:**
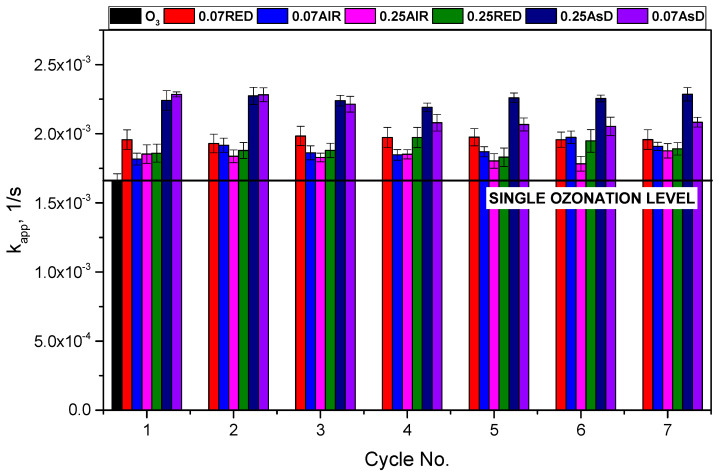
Kinetic constants of classical ozonation (pH 10) and heterogeneous catalytic ozonation (HCO) over W-based materials.

The kinetic analysis of the textile dye RB5 showed that all catalysts used in the experiment were active (Figure 3). The catalytic ozonation rates for all catalysts were higher than for single ozonation. In contrast, the kinetic constant for classical ozonation (pH 10) was as low as (1.66 ± 0.05) × 10^−3^ 1/s, while for the W catalyst (AsD, 80 W, *=* 0.25 Pa), it was (2.46 ± 0.03) × 10^−3^ 1/s.

Among the tested catalysts, the systems with AsD at Δ*p =* 0.25 Pa and Δ*p =* 0.7 Pa achieved the best results in terms of dye decolorization rates. However, the AsD Δ*p =* 0.7 Pa catalyst showed a decline in performance over consecutive cycles, indicating degradation or deactivation of the catalyst film. The AsD Δ*p =* 0.25 Pa catalyst also underwent some wear during the process. However, due to its thicker catalytic film, it maintained high efficiency over all 7 cycles tested. Over a longer period, its performance could begin to decline as the catalyst layer becomes consumed.

Interestingly, the catalysts post-treated in N_2_ and air atmospheres were less efficient but showed greater stability over time. This suggests that although they did not achieve the highest decolorization rates, their stability could make them suitable for applications requiring sustained catalytic activity.

**Figure 4:**
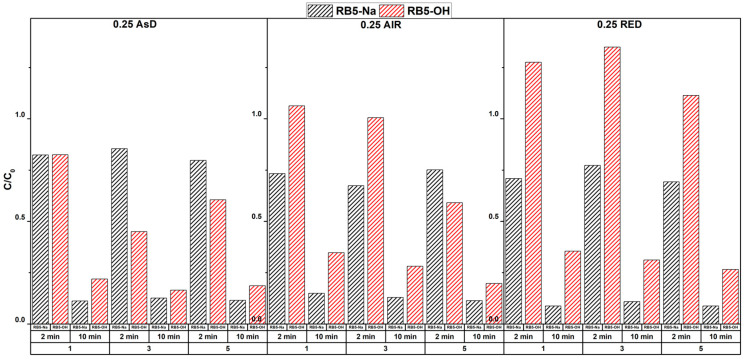
Relative concentration (C/C_0_) of Reactive Black 5 (RB5) dye in the form of sodium salt (RB5-Na) and hydrolyzed form (RB5-OH).

Figure 4 presents the ozonation process results for RB5 dye, analyzed in both its salt form (RB5-Na) and hydrolyzed form (RB5-OH) using high-pressure liquid chromatography (HPLC). The analysis evaluated the performance of three catalysts (for all thicker catalyst films, Δ*p =* 0.25). RB5-Na represents the parent dye, while RB5-OH is an early-stage by-product of RB5 decomposition. Both forms of the dye are colored, contributing to the overall decolorization rate depicted in Figure 3.

The RB5-Na concentration decreased consistently across all catalysts, with degradation varying depending on the catalyst type and ozonation time. For the AsD catalyst, the reduction in RB5-Na concentration was particularly significant after 10 min, showing a steady degradation trend. After 2 min, a relative concentration of 0.8 was observed for RB5-Na across each cycle. Notably, the RB5-OH form was more effectively decomposed during subsequent cycles with the AsD catalyst, suggesting a strong contribution from an adsorption-based mechanism. This effective decomposition of both RB5-Na and RB5-OH enhanced the overall decolorization rate.

For the Air post-treated catalyst (0.25 Air), high concentrations of the hydrolyzed RB5-OH form were observed, indicating it is a primary by-product of RB5-Na decomposition in the early stages. In subsequent cycles (e.g., cycle 5), a slight reduction in the decomposition rate was observed. However, after 10 min, performance remained high, surpassing the efficiency of the fresh catalyst. This demonstrates the durability of the Air catalyst over multiple uses. Both forms of the dye contributed to the decolorization process, and the transient accumulation of RB5-OH likely reflected the slower overall decolorization.

The N_2_ catalyst (0.25RED) exhibited a highly efficient decomposition of RB5-Na, leading to the production of RB5-OH, which was subsequently degraded at a moderate rate. This trend was consistent across cycles. However, the moderate rate of RB5-OH decomposition affected the overall decolorization efficiency, as shown in Figure 3. This suggests that the N_2_ catalyst effectively removed RB5-Na but was less efficient at rapidly degrading RB5-OH, prolonging its contribution to the overall coloration.

These observations highlight the critical roles of both RB5-Na and RB5-OH in the decolorization process. The performance differences between catalysts suggest distinct degradation mechanisms, with varying interactions between each catalyst and the parent dye (RB5-Na) as well as the hydrolyzed by-product (RB5-OH). The effectiveness of each catalyst was, therefore, determined not only by its ability to degrade RB5-Na but also by its efficiency in removing the colored RB5-OH intermediate, which impacted the decolorization rate shown in Figure 3.

### 2.3. Stability in Structure

**Figure 5:**
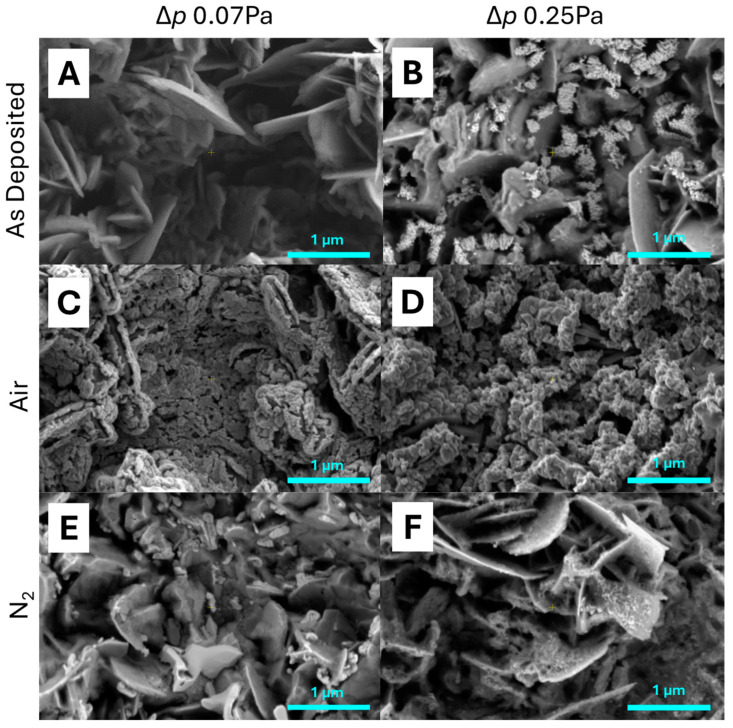
SEM images of microstructure after the catalytic process with application of W-based catalysts deposited through PECVD with 80 W power and two differential pressures of the precursor gas, and different post-treatment (AsD, Air, N_2_): Δ*p* = 0.07 Pa (**A**,**C**,**E**) and Δ*p* = 0.25 Pa (**B**,**D**,**F**).

Morphological changes after the catalytic process were visible in each sample, as shown in Figure 5. For the AsD samples, degradation of the layer occurred, revealing the plate-like Al_2_O_3_ substrate (Figure 5A,B). A residue of the layer was also visible in the 0.25 Pa sample, where distinct fragments of the layer appeared porous, with a size of about 100–200 nm in diameter (Figure 5B). The samples after calcination in air (Figure 5C,D) showed minimal but noticeable changes. The layer was more fragmented and porous, yet still adhered to the substrate surface. In contrast, the layers after pyrolysis in nitrogen (Figure 5E,F), following catalytic studies, had significantly degraded. A thin, delicate layer remained on the Al_2_O_3_ surface in the 0.25 Pa sample, while the 0.07 Pa sample displayed a completely different phase: solid precipitates, both large and small, on the alumina surface.

The atomic composition of the samples is summarized in Table 3. After the ozonation process, a significant loss of W was observed in all samples, with the least loss in the samples previously oxidized in air. Conversely, oxygen content increased in all samples to over 50%. The samples that underwent prior oxidation retained the least amount of carbon.

**Figure 6:**
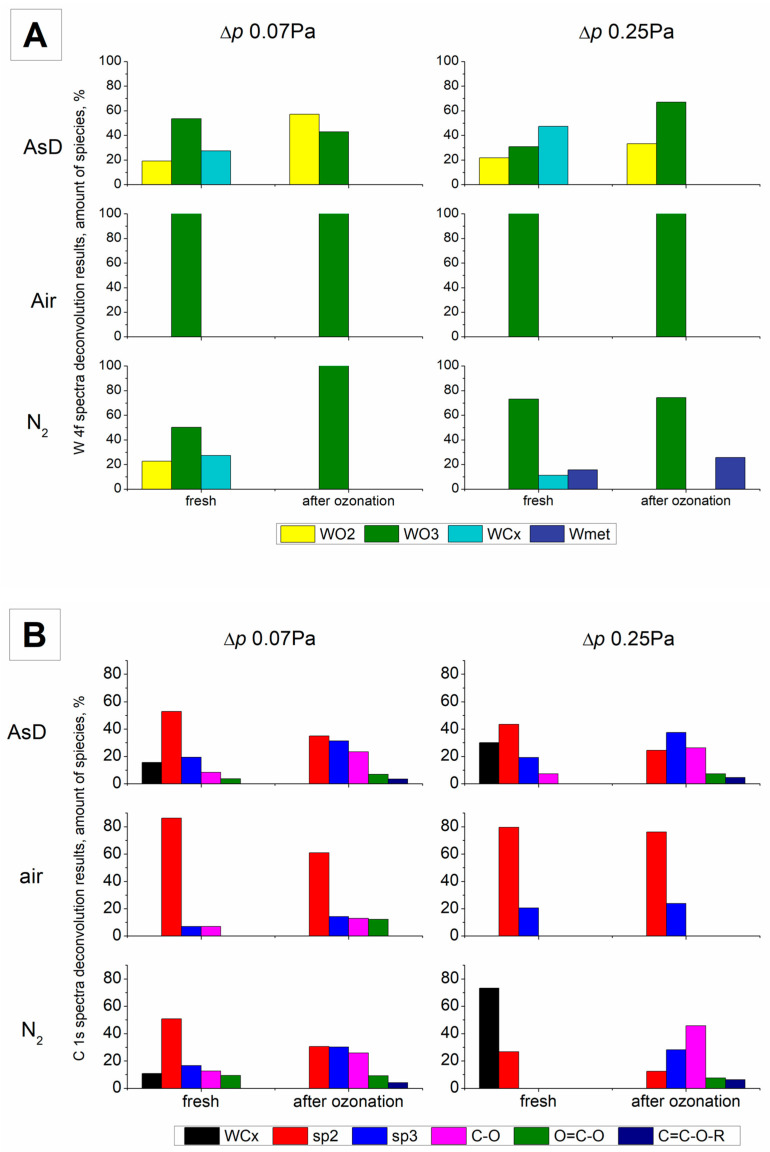
Summary of XPS deconvolution measurements for W4f (**A**) and C1s (**B**) positions for the analyzed samples before and after the catalytic process (ozonation).

A comparison of the changes in the samples, based on XPS measurements of the W4f and C1s spectra, is presented in Figure 6A,B, respectively. The results reveal that only the samples calcined in air remained largely unaffected by ozonation. These samples were composed entirely of W (VI) oxide, with no other W compounds detected.

Carbon analysis indicates that sp^2^ carbon was the predominant form in the samples, with a minor contribution from sp^3^ carbon. During ozonation, the carbon underwent slight oxidation. For the 0.07 Pa sample, this resulted in the formation of C–O and O=C–O bonds, along with sp^2^ carbon. In contrast, ozonation of the 0.25 Pa sample led to a slight increase in sp^2^ carbon content.

In the AsD samples, more pronounced changes were observed. WC, present in both samples (Figure 6A, light blue), was consumed during ozonation, leading to an increase in WO_2_. For the AsD *p* 0.07 sample, the amount of WO_2_ exceeded that of WO_3_ after ozonation, as WO_3_ content decreased. Conversely, the sample deposited at higher pressure exhibited a significant increase in WO_3_, which became more abundant than WO_2_.

Carbon spectrum analysis further confirmed the depletion of carbide during ozonation (Figure 6B, black). Additionally, the sp^2^ carbon content decreased, while the amounts of hydrocarbons (sp^3^) and oxidized carbon forms increased.

A similar pattern was observed for the N_2_ samples. In both cases, WC was consumed during ozonation. For the Δ*p* = 0.07 Pa sample, W was exclusively oxidized to WO_3_. In contrast, the higher-pressure sample behaved differently, with both WC and metallic W present. The proportion of metallic W increased following ozonation.

As with the AsD samples, carbon underwent oxidation. The sp^2^ carbon content decreased, W-bound carbon was eliminated, and hydrocarbons (sp^3^) and predominantly C–O bonds emerged.

### 2.4. Industrial Use

**Figure 7:**
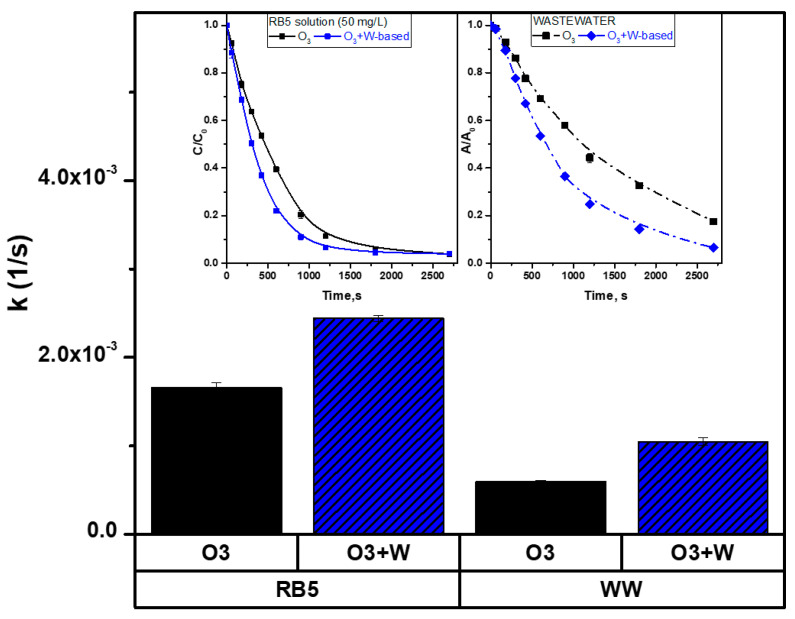
Kinetics of classical ozonation and HCO over W-based materials for RB5 solution and industrial textile wastewater (WW) containing the same dye.

The most active W-based catalyst, AsD Δ*p =* 0.25Pa, was used to decolorize real textile wastewater containing RB5. Its performance was compared with a test solution of the same dye under identical experimental conditions. The results showed that while catalytic efficiency for wastewater was lower, the same trend was observed: catalytic ozonation (O_3_ + W) significantly outperformed single ozonation (O_3_) in both cases (Figure 7).

For the test solution, the rate constant (k) was notably higher for catalytic ozonation compared to single ozonation, confirming the superior activity of the W catalyst AsD. Similarly, for wastewater, catalytic ozonation exhibited a much higher rate of decolorization than single ozonation. However, the overall efficiency for wastewater was lower due to the presence of additional pollutants and interfering substances, which competed for ozone and reactive oxygen species (ROS), reducing the rate of dye degradation.

These findings highlight that although real wastewater are demending due to complex composition, catalytic ozonation remained highly effective. The observed trend where catalytic ozonation outperformed single ozonation reinforces the robustness and applicability of the W catalyst, AsD, for both controlled test solutions and complex real wastewater matrices. This underscores the importance of catalytic ozonation as a promising approach for advanced wastewater treatment.

The COD removal was evaluated for RB5 decomposition in both simulated and industrial wastewater. The results of classical ozonation were compared with catalytic ozonation using a plasma catalyst. While the plasma catalyst promoted faster decomposition of by-products and may have facilitated more effective degradation pathways, the overall improvement in mineralization (as reflected by COD reduction) was only marginal.

For classical ozonation of RB5, the COD/COD₀ value decreased from 0.86 after 10 min to 0.50 after 45 min. In industrial wastewater, classical ozonation showed higher relative COD/COD₀ values of 0.97 after 10 min and 0.94 after 45 min, indicating lower efficiency in COD removal for more complex matrices.

Catalytic ozonation (ozone O_3_ and catalytically active 0.25 W AsD) of RB5 resulted in a slightly higher COD/COD₀ of 0.92 at 10 min and 0.56 at 45 min compared to classical ozonation. The slower COD reduction in the initial stages may suggest a difference in the reaction mechanism or the formation of intermediate products, which were degraded at a different rate. For industrial wastewater, catalytic ozonation achieved COD/COD_0_ values of 0.98 at 10 min and 0.90 at 45 min, showing minimal enhancement in COD removal compared to classical ozonation.

These results indicate that the catalytic process led to different final products compared to classical ozonation, as evidenced by the varying COD trends. While the plasma catalyst may have accelerated by-product decomposition, the formation of intermediate products during catalytic ozonation likely contributed to the slower reduction of COD values. This suggests that while catalytic ozonation offers potential benefits in altering degradation pathways and targeting specific pollutants, its effect on overall mineralization (reflected by COD reduction) remains limited for certain wastewater matrices.

## 3. Materials and Methods

### 3.1. Materials

The material of the support was wire (0.11 mm in diameter) made of Kanthal AF—FeCrAl alloy (TermTech, Warsaw, Poland), prepared by plasma etching and calcination as in [34]. A W hexacarbonyl (W(CO)_6_, (Merck, Darmstadt, Germany) was used as a precursor in PECVD for thin-film catalyst production. Wastewater for catalytic tests was prepared by dissolving textile RB5 dye (Boruta Zachem, Bydgoszcz, Poland) in distilled water with a pH 10 phosphate buffer: sodium dihydrogen phosphate (NaH_2_PO_4_) and sodium hydroxide (NaOH), both analytical grade (Chempur, Piekary Śląskie, Poland). The industrial wastewater, which originated from the dyeing of cotton fabric, was used in experiments. The dye and the auxiliaries of technical grade present in this wastewater included: (a) Remazol Ultraschwarz NN—industrial product based on RB5 (DyStar, Singapore); (b) Perigen LDR—industrial dyeing assistant SAA—a mixture of naphthalenesulfonic acid and carboxylates (Textilchemie Dr. Petry Co., Reutlingen, Germany); (c) NaCl (Solino, Inowrocław, Poland); (d) NaOH; and (e) Na_2_CO_3_ (Jarbur Eurochem, Szydłowiec, Poland). The wastewater was taken from an equalization reservoir (average wastewater from several dyeing operations). Table 4 presents the characteristics of the wastewater.

### 3.2. Catalyst Preparation

**Figure 8:**
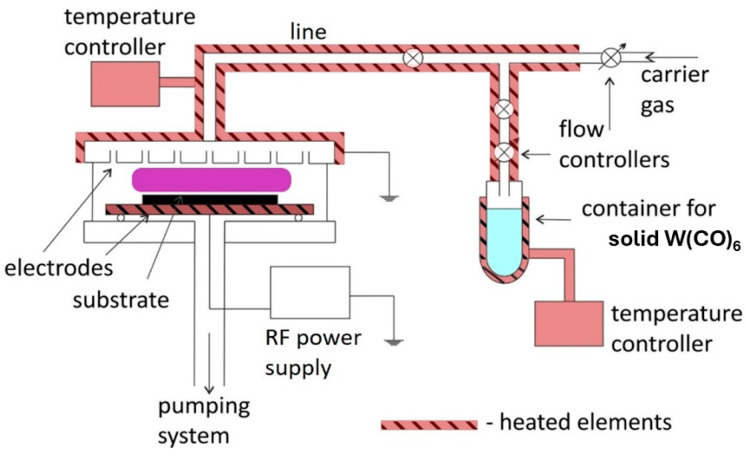
PECVD experimental set-up.

Low-temperature, non-equilibrium plasma was used for catalyst preparation under PECVD. A radio-frequency (RF 13.56 MHz) parallel-plate reactor was used for the process (a precise description of the reactor is presented elsewhere [34]). The PECVD operating conditions were as follows: glow discharge power 80 W, Ar carrier gas flow 2 sccm, W(CO)_6_ vapor pressure Δ*p* = 0.07 and 0.25 Pa, precursor temperature 80 °C, electrode temperature 120 °C, gas line temperature 120 °C, and deposition time 15 min. The experimental setup is depicted in Figure 8.

Two types of post-treatment were carried out. The first was air calcination at a temperature of 600 °C for 30 min (with a gradient of 15 °C per min). The second was nitrogen pyrolysis at a temperature of 850 °C for 30 min (with a gradient of 15 °C per min). Both were conducted in a tube furnace.

### 3.3. Catalytic Test

The semi-batch glass bubble column (31 cm high and 7 cm in diameter) with a stationary liquid phase of 1 L volume supported the catalytic experiment (HCO). The reactor was equipped with baffles of selected geometry (enabling the ozone flow), which served as carriers for the catalyst. A precise description is given under the patent claim P.445362 (European analog application EP23215146.4). Ozone gas, produced by the BMT 802N Generator (BMT Messtechnik GmbH, Stahnsdorf, Germany) from cylinder-compressed oxygen (O_2_ purity 99.5%) by Linde Gas (Łódź, Poland), was introduced into the system through a G3 ceramic frit placed at the reactor bottom. The gas-phase ozone concentration at the reactor inlet and outlet was monitored through the BMT 963 measurer (BMT Messtechnik GmbH, Stahnsdorf, Germany).

### 3.4. Analysis

The catalyst materials were analyzed using XPS with an AXIS Ultra spectrometer (Kratos Analytical Ltd., Manchester, UK) and monochromatic Al Kα X-rays (1486.6 eV). The anode power was set at 180 W, and the hemispherical electron energy analyzer operated at a pass energy of 20 eV for all high-resolution measurements. All measurements were carried out using a charge neutralizer, and the main carbon peak (graphitic C1s at 284.6 eV) was used for the final calibration of each spectrum.

The catalytic film morphology was investigated by SEM using an Apreo 2S microscope (Thermo Fisher Scientific, Waltham, MA, USA). Imaging was performed under an accelerating voltage of 2 kV using high-pressure mode. Samples were attached to carbon conductive tape and placed in the instrument’s analytical chamber. Samples were analyzed without coating.

The color was determined by a spectrophotometer Genesys 180 (Thermo Fisher Scientific, Waltham, MA, USA) through a calibration plot based on Lambert–Beer’s law conversion into concentration.

The COD indicator was detected by the abovementioned LCK cuvette tests (HACH), measured with a DR 3900 spectrophotometer (HACH, Loveland, CO, USA) following the manufacturer’s procedure.

The concentration of dye and by-products was investigated via HPLC (Model 1220 Infinity LC, Agilent Technologies, Santa Clara, CA, USA). HPLC was used to analyze dye solutions and intermediate products. A Zorbax Eclipse Plus C18 column (column size 50 mm × 4.6 mm, particle size 1.8 µm) with a UHPLC Guard 3PK pre-column (Agilent, Santa Clara, CA, USA) was used for the separation. The column temperature was set to 30 °C. The mobile phase was a mixture of water acidified with 0.1% formic acid (phase A) and acetonitrile (phase B), with a flow rate set at 0.4 mL/min. The gradient was set as follows: 90% A from 0.00–6.00 min, 0% A from 6.00–7.00 min, 90% A from 7.30–9.00 min, 0% A from 10.00–12.00 min, 90% A at 12.00 min. A 3-min post-time was set between measurements. The wavelength of the UV/Vis detector (diode array detector) was set to 598 nm.

## 4. Conclusions

The activity of the studied catalysts is strongly dependent on the presence of carbon in their structure, as confirmed by XPS analysis. The XPS results revealed a shift in carbon hybridization from sp^2^ to sp^3^, suggesting oxidation processes occurring on the catalyst surface during the reactions.

Carbon in the sp^2^ state, typical of delocalized π-electron systems (e.g., graphene or aromatic structures), is associated with high catalytic activity. However, the observed transition to sp^3^ indicated the formation of more saturated carbon structures, likely due to surface oxidation. This process may introduce oxygen-containing functional groups (e.g., hydroxyl or carbonyl groups), which can alter the catalyst’s performance.

Although these changes can enhance adsorption properties and reaction efficiency in the short term, the reduction of active sp^2^ carbon over time may lead to decreased catalytic activity. Therefore, controlling carbon oxidation and maintaining a balance between sp^2^ and sp³ states is crucial for ensuring long-term catalyst stability and efficiency.

The XPS analysis confirmed that catalytic activity is closely linked to carbon hybridization, with the shift from sp^2^ to sp^3^ highlighting the impact of oxidation on catalyst performance.

Additionally, the presence of WC likely plays a significant role in the catalytic activity during ozonation. WC is known for its high surface reactivity and ability to facilitate electron transfer processes. Its interaction with ozone may enhance the generation of ROS, such as hydroxyl radicals, which are essential for the degradation of organic pollutants. Furthermore, the synergistic effect between WC and the carbon matrix may improve the overall catalytic performance by providing active sites for ozone adsorption and activation, while also enhancing the catalyst’s stability under oxidative conditions.

While WO_3_ is typically active in photocatalytic systems, it appears to play a secondary role in this case. The catalytic mechanism involving the formation of ozonide radicals (O_3_^−^) seems to have limited significance here, as it is more relevant in WO_3_-dominated processes under neutral or acidic conditions [14]. These radicals, though potent oxidizing species, are not the primary contributors to the observed catalytic activity in this system.

The observed differences in catalyst performance highlight distinct degradation mechanisms and variations in interactions with both the parent dye (RB5-Na) and the hydrolyzed by-product (RB5-OH), as shown in Figure 4. The catalytic activity is closely linked to carbon hybridization, with the sp²-to-sp³ transition and WC playing critical roles in reaction efficiency. The balance between sp^2^ and sp^3^ carbon, along with the synergistic interactions between WC and the carbon matrix, is key to maintaining high catalytic activity and ensuring effective dye decolorization.

In conclusion, this study demonstrates the successful design and preparation of W-based catalysts with varied morphologies and compositions, achieved through precise control of PECVD parameters. The results highlight the catalysts’ exceptional stability and reusability over multiple cycles of catalytic ozonation, emphasizing their potential as sustainable and efficient solutions for textile wastewater treatment with enhanced operational durability and consistent color removal efficiency.

## 5. Patents

The construction of the bubble column and catalyst usage are covered under the Polish patent claim application (P.445362) and the European consolidated patent claim (analog application EP23215146.4).

## Figures and Tables

**Table 1:** XPS atomic composition of prepared samples.

Sample	Atomic Composition, %						Ratio		
	W		C		O		W/C	W/O	C/O
0.07 AsD	13.65	±0.95	55.87	±2.12	30.47	±1.18	0.24	0.45	1.83
0.07 N_2_	15.38	±0.75	48.62	±3.46	36.00	±3.36	0.32	0.43	1.35
0.07 Air	13.00	±0.41	41.37	±2.26	45.64	±2.25	0.31	0.28	0.91
0.25 AsD	23.47	±1.79	43.00	±3.79	33.52	±2.43	0.55	0.70	1.28
0.25 N_2_	17.40	±0.25	27.50	±2.43	55.10	±2.26	0.63	0.32	0.50
0.25 Air	17.56	±0.36	21.11	±0.99	61.23	±1.13	0.83	0.29	0.34

**Table 2:** Summarized XPS results from deconvolution of W and C for prepared samples.

	0.07 Pa			0.25 Pa		
	AsD	Air	N_2_	AsD	Air	N_2_
	W4f deconvolution					
WO_2_, %	19.2	0	22.6	21.8	0	0
WO_3_, %	53.5	100	50.2	30.9	100	73.2
WCx, %	27.4	0	27.2	47.3	0	11.2
Wmet, %	0	0	0	0	0	15.6
	C1s deconvolution					
WCx	15.6	0	10.8	30.1	0	73.3
sp^2^	52.9	86.3	50.7	43.4	79.5	26.7
sp^3^	19.4	6.9	16.6	19.2	20.5	0
C–O	8.4	6.9	12.6	7.3	0	0
O=C–O	3.7	0	9.3	0	0	0
C=C–O–R	0	0	0	0	0	0

**Table 3:** XPS atomic composition of samples after catalytic process.

Sample	Atomic Composition, %						Ratio		
	W		C		O		W/C	W/O	C/O
0.07 AsD-ozonated	0.15	±0	38.32	±0.59	63.20	±3.7	0.00	0.00	0.61
0.07 N_2_-ozonated	1.88	±0.1	40.68	±0.5	57.43	±0.39	0.05	0.03	0.71
0.07 Air-ozonated	10.47	±0.23	33.42	±2.26	56.11	±2.16	0.31	0.19	0.60
0.25 AsD-ozonated	2.31	±0.17	45.44	±0.65	52.25	±0.48	0.05	0.04	0.87
0.25 N_2_-ozonated	2.50	±0.04	44.79	±1.02	52.70	±1.06	0.06	0.05	0.85
0.25 Air-ozonated	13.54	±0.04	25.11	±0.96	61.35	±0.97	0.54	0.22	0.41

**Table 4:** Textile wastewater parameters.

Parameter	Value, mg/L
Dye concentration (RB5)	750
NaCl concentration	75,000
pH	10.6
COD	3440
TOC *	1790

* Total Organic Carbon.

## Data Availability

Data are available upon request.
